# Effects of *Lactobacillus plantarum* FZU3013-Fermented *Laminaria japonica* on Lipid Metabolism and Gut Microbiota in Hyperlipidaemic Rats

**DOI:** 10.3389/fnut.2021.786571

**Published:** 2021-12-06

**Authors:** Jin-Peng Hu, Ting-Ting Zheng, Bin-Fen Zeng, Man-Ling Wu, Rui Shi, Ye Zhang, Li-Jiao Chen, Wen-Jian Cheng, Peng Liang

**Affiliations:** College of Food Science, Fujian Agriculture and Forestry University, Fuzhou, China

**Keywords:** *Laminaria japonica*, *Lactobacillus plantarum* FZU3013, hyperlipidaemia, lipid metabolism, gut microbiota

## Abstract

In this study, we explored the effect of *Lactobacillus plantarum* FZU3013-fermented *Laminaria japonica* (LPLJ) supplementation to prevent hyperlipidaemia in rats fed with a high-fat diet (HFD). The results indicate that LPLJ supplementation improved serum and hepatic biochemical indicators (*p* < 0.05), elevated short-chain fatty acid levels, reduced HFD-induced accumulation of lipid droplets in the liver, modulated the relative abundance of some microbial phylotypes, and reduced hyperlipidaemia in HFD-fed rats by adjusting the aminoacyl-tRNA, phenylalanine, tyrosine, and tryptophan biosynthetic pathways, as well as the phenylalanine, D-glutamine and D-glutamate, and glutathione metabolic pathways. Additionally, hepatic mRNA levels of the genes involved in lipid metabolism and bile acid homeostasis were significantly reduced by LPLJ intervention (*p* < 0.05). These results suggest that LPLJ has a positive effect on modulating lipid metabolism and has the potential to be a functional food that can help prevent hyperlipidaemia.

## Introduction

The incidence of hyperlipidaemia is rapidly increasing owing to improved socio-economic status and consumption of unhealthy diets. Hyperlipidaemia is linked to the development of cardiovascular diseases, resulting in non-alcoholic fatty liver (NAFL) to a certain extent, and this has received considerable critical attention (1–4). The main treatment for hyperlipidaemia is drug therapy, which often leads to various adverse reactions (5). Currently, researchers are focusing their attention on food therapy to prevent hyperlipidaemia because of its high efficacy complemented with few or no side effects.

*Laminaria japonica* (LJ) has diverse bioactive compounds, such as proteins, polysaccharides, and vitamins (6, 7). Polysaccharides are an important active constituent of LJ and have a variety of physiological functions, including hypolipidemic capacity, bile acid (BA)-binding, anti-atherosclerosis, and anti-bacterial activities (8–10). With the development of microbial fermentation technology, many researchers have applied this technology to the processing and development of LJ-containing foods. The fermentation of lactic acid bacteria (LAB) can promote the transformation of nutrients and hydrolysis of biological macromolecules, as well as increase the content of bioactive substances. As the most commonly used probiotics in food fermentation, LAB have been shown to relieve hyperlipidaemia. For example, *Lactobacillus plantarum* regulates dyslipidaemia, the intestinal microbiome, and hepatic metabolism in high-fat diet (HFD)-induced hyperlipidaemic rats (11). However, the regulatory mechanism of *L. plantarum-*fermented LJ (LPLJ) on hyperlipidaemia is not yet known.

Previous studies have shown that lipid metabolism in the liver is one of the most critical factors in maintaining lipid metabolism and homeostasis (12, 13). The synergistic changes in the transport of lipoprotein and the absorption, biosynthesis, and catabolism of cholesterol in the liver are closely correlated with the modulation of lipid metabolism (14). In addition, as a significant pathway of liver lipid metabolism, enterohepatic circulation is pivotal to human health. The major factors influencing the composition of gut microbiota are diet (15), lifestyle (16), mood (17), and antibiotic treatment (18). Clinical research has shown that the consumption of high-fat food for a long period results in the reduction of gut microbial diversity (19). Emerging evidence indicates that intestinal flora dysbiosis might result in obesity (20), hyperlipidaemia (21), and NAFL (22). To date, the impact of LPLJ on lipid metabolism in the liver and its relationship with intestinal flora regulation have not been reported.

Metabolomic analysis can detect changes in metabolism under different stimuli by analyzing the changes in endogenous small molecule metabolites (23). A previous study applied ultra-high performance liquid chromatography-quadrupole time-of-flight mass spectrometry (UPLC-QTOF/MS) for serum metabolomics and showed that the levels of amino acids and BAs are closely related to the occurrence and development of hyperlipidaemia (24, 25). However, there have been no reports on the mechanism of action of LPLJ supplementation in liver metabolomics based on UHPLC-QTOF/MS in patients with hyperlipidaemia.

Therefore, liver metabolomics and high-throughput sequencing of the gut microflora were used in this study to investigate the effect of LPLJ on lipid metabolism and determine its lipid-lowering mechanism in hyperlipidaemic rats. The findings of this study will provide useful information to identify functional foods that can prevent hyperlipidaemia.

## Materials and Methods

### Preparation of *L. plantarum* FZU3013-Fermented LJ

*L. plantarum* FZU3013, isolated from the fermentation of *Hongqu* rice wine, was obtained from the Institute of Food Science and Technology, Fuzhou University (6, 26). This strain was cultured in the MRS medium for 36 h under static conditions, and then sub-cultured three times. Fermentation of LJ was conducted according to a previously described method (27). LJ was purchased from the Yonghui supermarket (Fuzhou, China). Briefly, LJ was washed, dried, and crushed (80 mesh). The powder (3 g) was dissolved in 100 mL of deionised water, autoclaved at 110 °C for 15 min, and, after cooling, inoculated with 1 mL of a cell suspension (1.0 × 10^9^ CFU/mL) of *L. plantarum* FZU3013. Then, the mixture was fermented at 37 °C for 24 h.

### Measurement of Chemical Characteristics

The total sugar content in LJ before and after fermentation was determined by the phenol-sulfuric method. The sample was centrifuged (8,000 × g, 15 min), and 1 mL of the supernatant was mixed with 1 mL of 5% phenol solution, and then 6 mL of concentrated sulfuric acid was added. The reaction mixture was left standing at room temperature for 30 min. The absorbance was measured at 490 nm with D-glucose as a standard. The reducing sugar content was determined by the dinitrosalicylic acid (DNS) method. Briefly, 1 mL of sample supernatant was mixed with 2 mL DNS (3, 5-dinitrosalicylic acid, 2M NaOH, and potassium sodium tartrate) in boiling water for 15 min, and the absorbance was measured at 540 nm. The total polyphenol content was measured using a total phenol content detection kit (Beijing Solaibao Science & Technology Co., Ltd). In short, 3 mL of the sample was mixed with 2.5 mL of 60% ethanol and extracted using ultrasound (300 W, 60°C, 30 min). After centrifugation (8,000 × g, 25 °C, 10 min), the supernatant was collected, and the absorbance was measured at 760 nm with gallic acid as the standard compound.

### Animal Experiments

Forty male specific pathogen-free rats (6 weeks of age) were purchased from Sipeifu Biotechnology Co., Ltd. (Beijing, China) and maintained under a specific environment (temperature 22 ± 4 °C, humidity 60% ± 10%, 12-h light/dark cycle). After a week of acclimation, the rats were randomly split into five groups (n = 8): (1) a normal-fat diet (NFD), (2) an HFD, (3) an HFD with 10 mg/kg simvastatin (Simv) per day, (4) an HFD with 2.0 g/kg LJ powder suspension per day, and (5) an HFD with 2.0 g/kg LPLJ per day.

### Biochemical Analysis of Serum and Liver

At the 8th week of these experiments, rats were euthanized after overnight fasting. Blood samples were collected from the heart into 2-mL centrifuge tubes and incubated at 25 °C for 1 h. Serum was obtained by centrifugation (3,000 × *g*, 8 min). Serum total cholesterol (TC), triglyceride (TG), and non-esterified fatty acid (NEFA) levels were measured using rapid detection kits (Kilton Biotechnology Co., Ltd., Shanghai, China). The liver samples were dispersed in a saline solution (1: 9) and then homogenized. The sediment was removed by centrifugation at 3,000 × *g* for 10 min. TC, TG, NEFA, and total BA (TBA) levels in the livers were also determined using commercial kits (Kilton Biotechnology Co., Ltd., Shanghai, China). Fecal TBA, TC, and TG levels were measured using kits from Kilton Biotechnology Co., Ltd.

### Histopathologic Evaluation

Fresh liver or adipose tissue samples were fixed in formalin solution (4%) overnight. Tissue sections (5 μm) were prepared, stained with haematoxylin and eosin (5), and examined under light microscopy (Bresser, Borken, Germany).

### Determination of Fecal Short-Chain Fatty Acids (SCFAs)

SCFAs were analyzed by the method described by Guo et al. (28). In brief, feces (50 mg) were diluted with 500 μL of saturated NaCl solution and incubated at 25 °C for 30 min. The mixture was then homogenized for 3 min, followed by the addition of 20 μL of 10% sulphuric acid solution and shaking for 30 s. Subsequently, 800 μL of diethyl ether and 20 μL of 10% sulphuric acid solution were added. Total SCFAs were extracted by centrifugation (4 °C, 8,000 × *g*, 15 min). To remove trace water, 0.25 g of Na_2_SO_4_ (anhydrous) was added to the supernatant. After sequential centrifugation (3,000 × *g*, 4 °C, 1 min) and filtration through a 0.22-μm filter, the extracted fecal SCFAs were evaluated using capillary gas chromatography.

### High-Throughput Sequencing of Gut Microbiota

Genomic DNA was extracted from cecum samples using a fecal DNA isolation kit (MoBio, USA). Bacterial full-length 16S rDNA was amplified by PCR with primers 338F/806R. The purified PCR products were used to construct DNA libraries with the Pacific Biosciences Template Prep Kit 2.0 (Pacific Biosciences). The high-throughput sequencing was performed on the PacBio RS II platform at Shanghai Personal Biotechnology Co., Ltd. (Shanghai, China). Sequences were classified as operational taxonomic units with a similarity threshold of 97%. Significant differences in gut microbial phylotypes between different groups were demonstrated by STAMP (Ver. 2.1.3). The R software (Ver. 3.3.3) was used to draw a heat map of the correlation between lipid metabolism and key intestinal microbial phylotypes. The correlation network was visualized by Cytoscape (Ver. 3.6.0).

### UPLC-QTOF/MS-Based Liver Metabolomics

Fifty milligrams of specimen (liver lobe) was added to an Eppendorf tube, and 1,000 μL of extracting solvent was added. After 30 s of vortexing, the mixture was homogenized in an ice water bath with an ultrasonic instrument for 4 min at a frequency of 35 Hz. The specimens were incubated at−40 °C for 1 h and centrifuged (8,000 × g, 4 °C, 15 min). The detailed operating parameters of UPLC-QTOF/MS were performed as described in a previous study (29). The mobile phase consisted of 25 mmol/L ammonium acetate and 25 mmol/L ammonia hydroxide in water (pH = 9.75) (A) and acetonitrile (B). The analysis was carried out with an elution gradient as follows: 0–1.0 min, 95 % B; 1.0–8.0 min, 95–65% B; 8.0–9.0 min, 65–40% B; 9.0–10.0 min, 40% B; 10.0–10.2 min, 40–95% B; and 10.3–12.0 min, 95% B.

### Real-Time Quantitative PCR

The liver total RNA was extracted according to the kit instructions and reverse-transcribed into cDNA by the PrimeScript™RT reagent kit (Takara, Japan). The sequences of primers are listed in Table 1. qRT-PCR was performed in StrataGene Mx3005P (Agilent, Colorado, USA) in combination with SYBR Premix Ex Taq II (Takara, Dalian, China). The mRNA expression level was normalized to that of β-Actin as a reference. The combination of experimental data was expressed as the mean ± standard deviation (SD).

**Table 1:** Primer sequences for quantitative real-time PCR.

**Gene**	**Forward primer (5'-3')**	**Reverse primer (5'-3')**
HMGCR	AGTGGTGCGTCTTCCTCG	CGAATCTGCTGGTGCTAT
SREBP-1C	GCTGTTGGCATCCTGCTATC	TAGCTGGAAGTGACGGTGGT
CYP7A1	CTGCGAAGGCATTTGGACACAGA	GCATCTCCCTGGAGGGTTTTGGT
ACOX1	TTACATGCCTTTGTTGTCCCTATC	CGGTAATTGTCCATCTTCAGGTA
ACAT2	GAACGTGGTGGTCCATGACT	TTCAGCAGACCTCCAACCAC
BSEP	CGTGCTTGTGGAAGAAGTTG	GGGAGTAGATGGGTGTGACTG
CD36	GACAATCAAAAGGGAAGTTG	CCTCTCTGTTTAACCTTGAT
LDLR	ATGCTGGAGATAGAGTGGAGTT	CCGCCAAGATCAAGAAAG

### Statistical Analysis

All data are expressed as the mean ± SD. Statistical differences were analyzed using GraphPad Prism 7.0 for one-way ANOVA with Tukey's comparisons test (*p* < 0.05). The significance levels of test were set at *p* < 0.05.

## Results

### Changes in Chemical Characteristics

Changes by fermentation are shown in Table 2. The log CFU of LJ after fermentation was 8.89 ± 0.02, and the pH decreased significantly compared with before fermentation (*p* < 0.05). After 24 h of fermentation, the total sugar was obviously decreased (*p* < 0.05). Reducing sugar and polyphenols were not significantly decreased.

**Table 2:** Changes of pH, CFU, total sugars, reducing sugars, and total phenolic compounds by fermentation.

**Sample**	**pH**	**Log CFU/mL**	**Total sugars (mg/mL)**	**Reducing sugars (mg/mL)**	**Total phenolic compounds (μg gallic acid/ML)**
**Before (LJ)**	6.42 ± 0.12	\	0.39 ± 0.02	0.12 ± 0.01	1.11 ± 0.02
**After (LPLJ)**	4.03 ± 0.09^a^	8.32 ± 0.02	0.26 ± 0.03 ^a^	0.14 ± 0.01	0.97 ± 0.04

a *Values are mean ± standard deviation (n = 3; p < 0.05, significantly different from the LJ group)*.

### Effects of LPLJ on Physiological Indices

**Figure 1:**
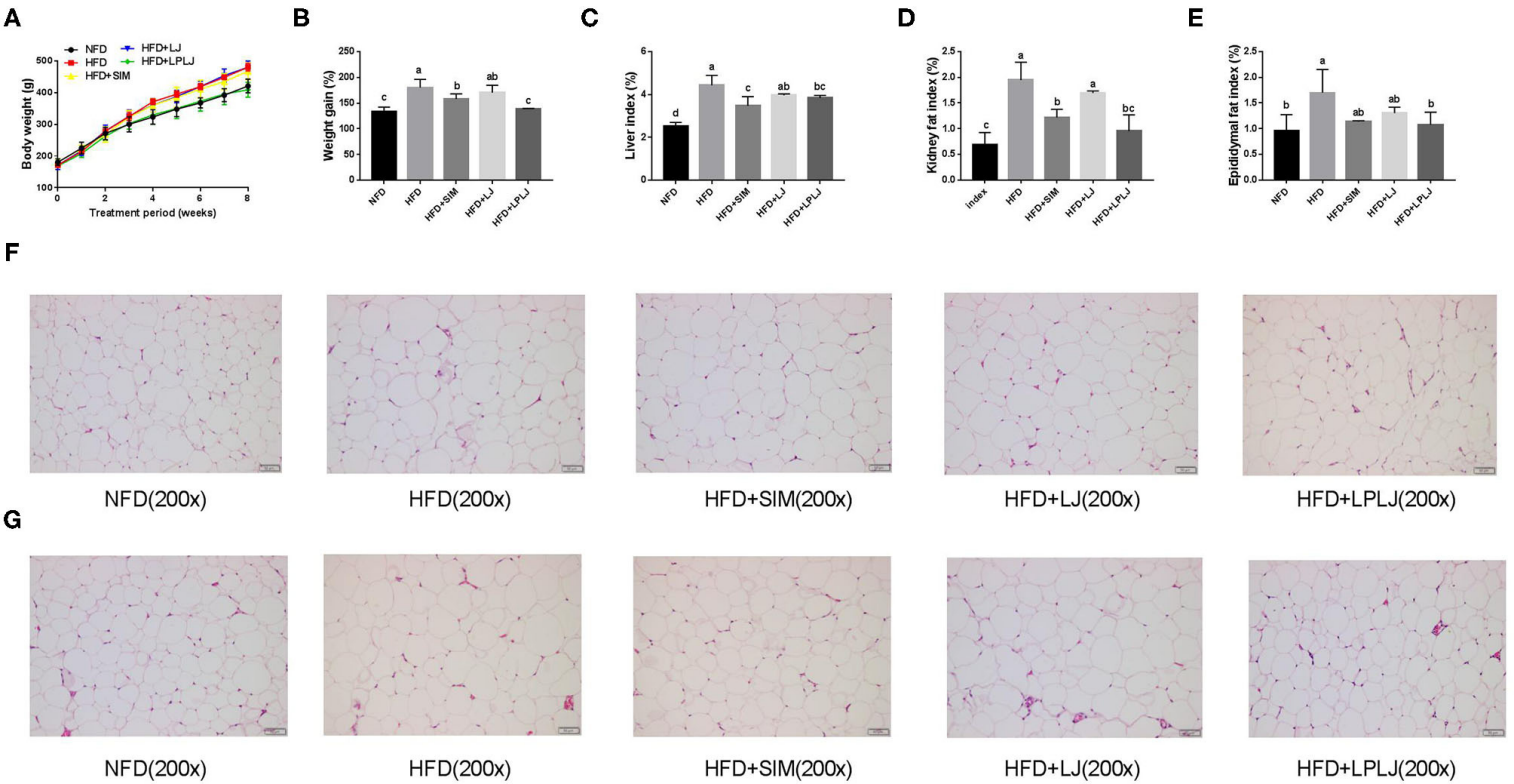
Effects of LPLJ consumption on **(A)** body weight, **(B)** weight gain, **(C)** liver index, **(D)** kidney fat index, **(E)** epididymal fat index, **(F)** the size of perirenal adipocytes and **(G)** the size of epididymal adipocytes in rats fed a high fat diet. a-c, Mean values with different letters in the same column differ significantly (*p* < 0.05).

After 8 weeks of feeding, the body weight of the HFD-fed rat group was markedly higher than that of the NFD group (Figures 1A,B). The increasing trend caused by the HFD was reduced by dietary supplementation with LPLJ. In addition, HFD-fed rats showed an evident increase in the levels of physiological indices of the liver, perirenal adipocytes, and epididymal adipocytes compared with NFD-fed rats (Figures 1C–E). However, these increased levels were significantly alleviated by dietary supplementation with LPLJ (*p* < 0.05). Perirenal and epididymal adipocytes were with smaller size and volume in the other three groups than in the HFD group (Figures 1F,G) (*p* < 0.05).

### Effects of LPLJ on Biochemical Indicators of Blood Serum

**Figure 2:**
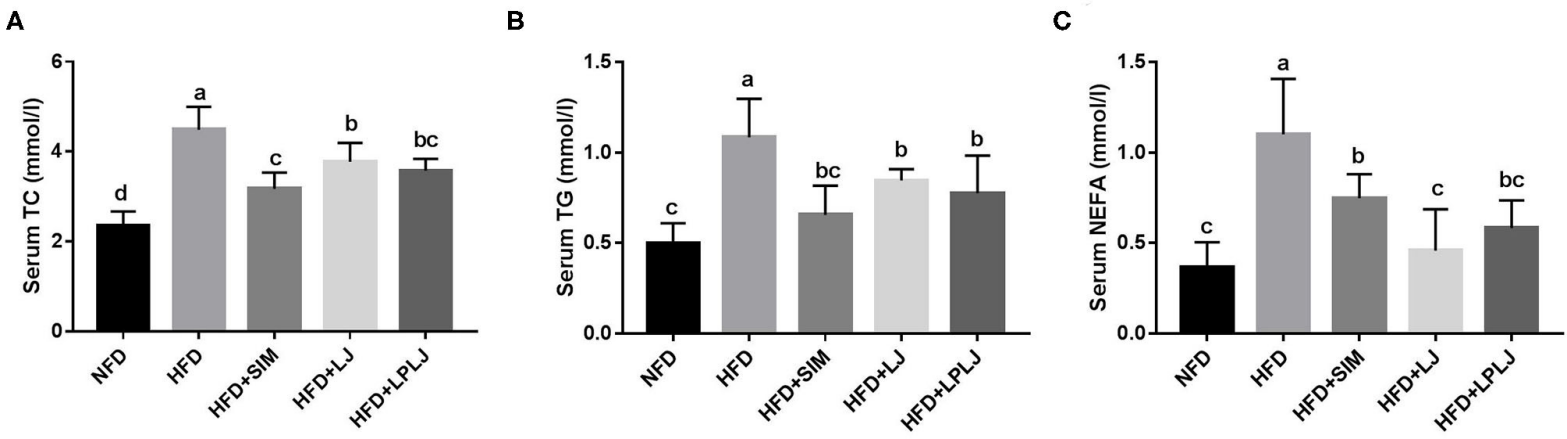
Effects of LPLJ consumption on the serum biochemical parameters in rats fed a high-fat diet for consecutive 8 weeks. **(A)** serum TC, **(B)** serum TG, and **(C)** serum NEFA. a-d, Mean values with different letters in the same column differ significantly (*p* < 0.05).

After 8 weeks of treatment, serum TG, TC, and NEFA levels were effectively increased in rats in the HFD group compared with those in the NFD group (Figure 2). Dietary supplementation with LPLJ effectively reduced the serum TC, TG, and NEFA levels that were increased by the HFD (*p* < 0.05).

### Effects of LPLJ on Liver Biochemical Indices and Histopathology

**Figure 3:**
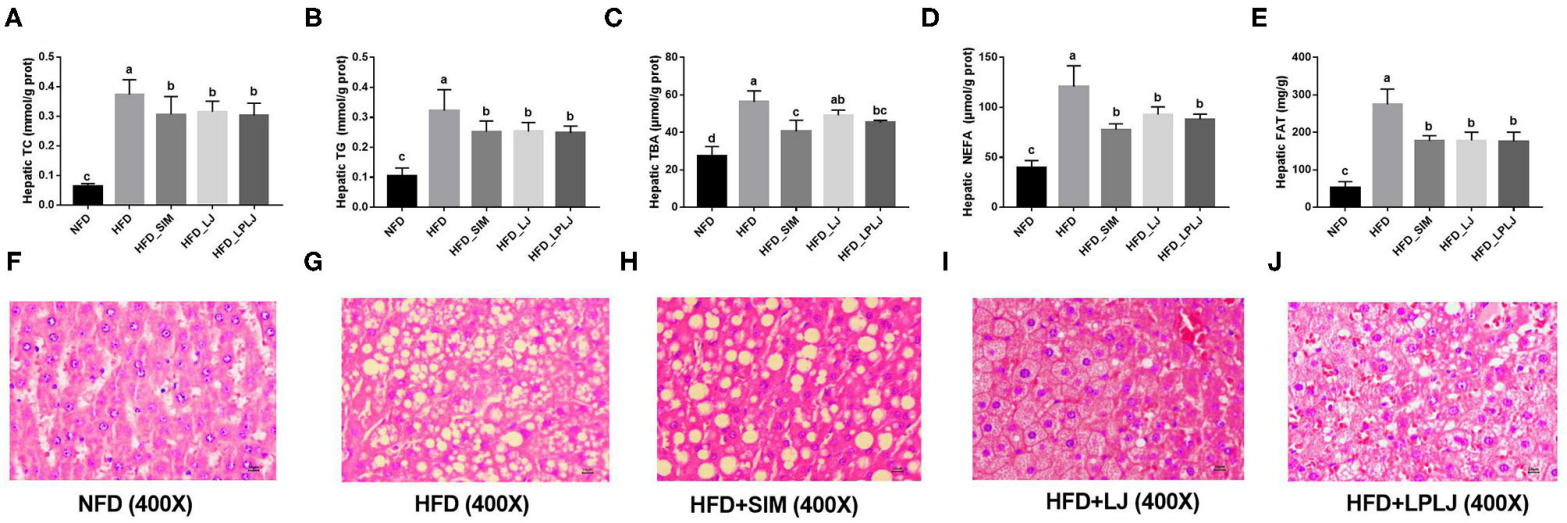
Effects of LPLJ consumption on the liver biochemical parameters and histopathology in rats fed a high-fat diet for consecutive 8 weeks. **(A)** hepatic TC, **(B)** hepatic TG, **(C)** hepatic TBA, **(D)** hepatic NEFA, **(E)** hepatic FAT, and **(F–J)** liver morphology (magnification × 400) in rats fed a high fat diet. a-d, Mean values with different letters in the same column differ significantly (*p* < 0.05).

To determine the potential regulatory effects of LPLJ on lipid accumulation, the hepatic TG, TC, TBA, and NEFA levels of HFD-fed rats were determined. HFD-fed rats showed higher hepatic TG, TC, TBA, NEFA, and FAT levels (Figures 3A–D). Similar to the effect of Simv, the oral administration of LPLJ significantly prevented these adverse changes caused by the HFD (*p* < 0.05). Dietary supplementation with LPLJ was observed to reduce the hepatic TG, TC, TBA, and NEFA levels. Moreover, liver fat levels in the HFD group were markedly decreased by the oral administration of LPLJ (Figure 3E) (*p* < 0.05). Furthermore, histological analysis demonstrated that lipid droplet accumulation was markedly increased in HFD-fed rats. Notably, the size and quantity of fat vacuoles in the LPLJ group were much lower than those in the HFD group (Figures 3F–J). The results suggest that supplementation with Simv, LJ, and LPLJ effectively reduces hepatic lipid accumulation in hyperlipidaemic rats.

### Effects of LPLJ on the Level of Fecal Lipid and SCFAs in HFD-Fed Rats

**Figure 4:**
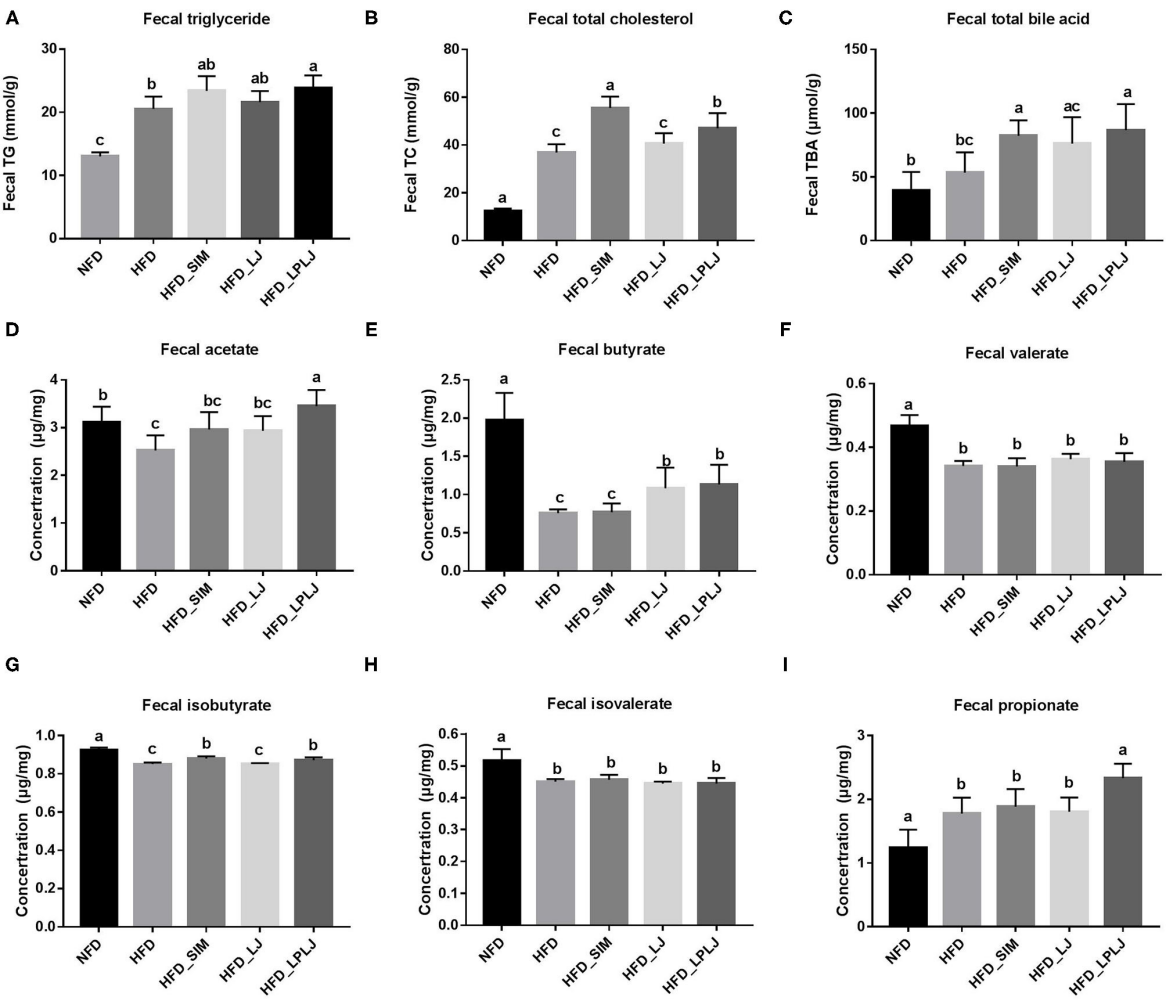
Effect of LPLJ consumption on the fecal lipid levels and short-chain fatty acids (SCFAs) in rats fed a high fat diet. **(A)** TG, **(B)** TC, **(C)** TBA, **(D)** fecal acetate, **(E)** fecal butyrate, **(F)** fecal valerate, **(G)** fecal isobutyrate, **(H)** fecal isovalerate, and **(I)** fecal propionate levels. a-c, Mean values with different letters in the same column differ significantly (*p* < 0.05).

Fecal levels of TBA, TG, and TC were increased by LPLJ diet intervention compared with those in the HFD group (Figures 4A–C) (*p* < 0.05). The results suggest that daily supplementation with LPLJ effectively increases the fecal excretion of lipids. In addition, compared with the NFD group, the fecal acetate, butyrate, isobutyrate, valerate, and isovalerate levels were reduced by high-fat feeding (Figures 4D–G) (*p* < 0.05). However, supplementation with LPLJ remarkably increased the acid acetate, butyrate, propionate, and isobutyrate levels (Figures 4H,I) (*p* < 0.05).

### LPLJ Supplementation Regulated the Intestinal Microbiota

**Figure 5:**
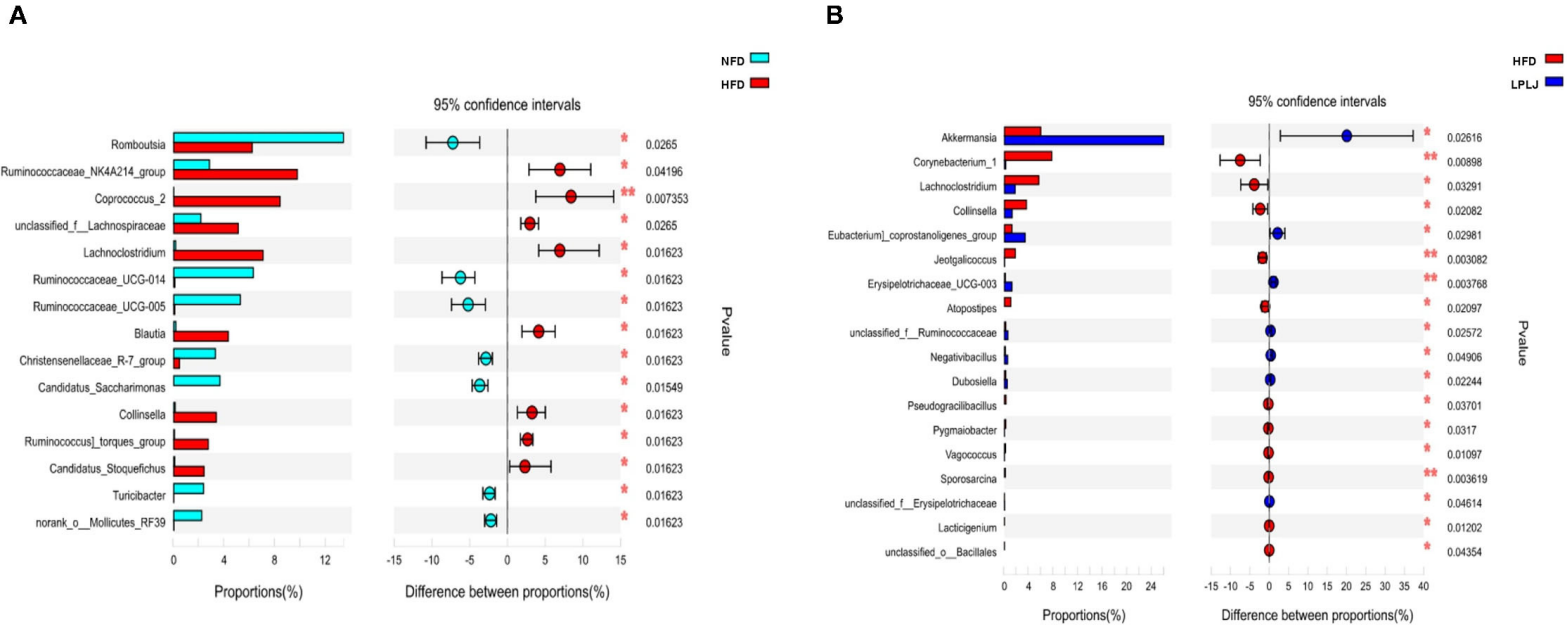
Extended error bar plot comparing the differences in the mean proportions of the significantly altered intestinal microbial phylotypes. The differences between groups were determined using a Welsh's *t*-test, and the Benjamini-Hochberg procedure was used to control the false-discovery rate due to multiple testing. Corrected *P* values are shown at right. **(A)** NFD group vs. HFD group, **(B)** HFD+LPLJ group vs. HFD group. Values were expressed as mean ± SEM in each group (*n* = 8). *p < 0.05, **p < 0.01.

To evaluate the influence of LPLJ on intestinal microbial composition, the V3–V4 region of the HFD+LPLJ group was sequenced using high-throughput sequencing. Key microbial system types with significant differences between the NFD (cyan) and HFD (red), and HFD (red) and HFD+LPLJ (blue), were revealed using the STAMP software (Figure 5). Compared with that in the NFD group, the relative richness of 15 key microbial system types was markedly different in the HFD group; that of eight system types was significantly increased and that of seven system types was significantly decreased (Figure 5), indicating that changes in intestinal microflora occurred in HFD-induced hyperlipidaemic rats. However, supplementation with LPLJ effectively modified the intestinal microbiota induced by the HFD. Supplementation with LPLJ increased the relative abundance of *Akkermansia, Eubacteriumcoprostanoligenes_group, Erysipelotrichaceae_UCG-003, unclassified_f_Ruminooccaceae, Negativibaillus, Dubosiella*, and *unclassifed_f_Erysipelotrichaceae*, but decreased that of *Corynebacterium_1, Lachnoclostridium, Collinsella, Jeotgalcoccus, Atopostipes, Pseudograllibacllus, Pygmaiobacter, Vagooccus, Sporosarcina*, and *Lacticigenium*.

### Correlations Between Key Intestinal Microflora Phylotypes and Lipid Metabolic Parameters

**Figure 6:**
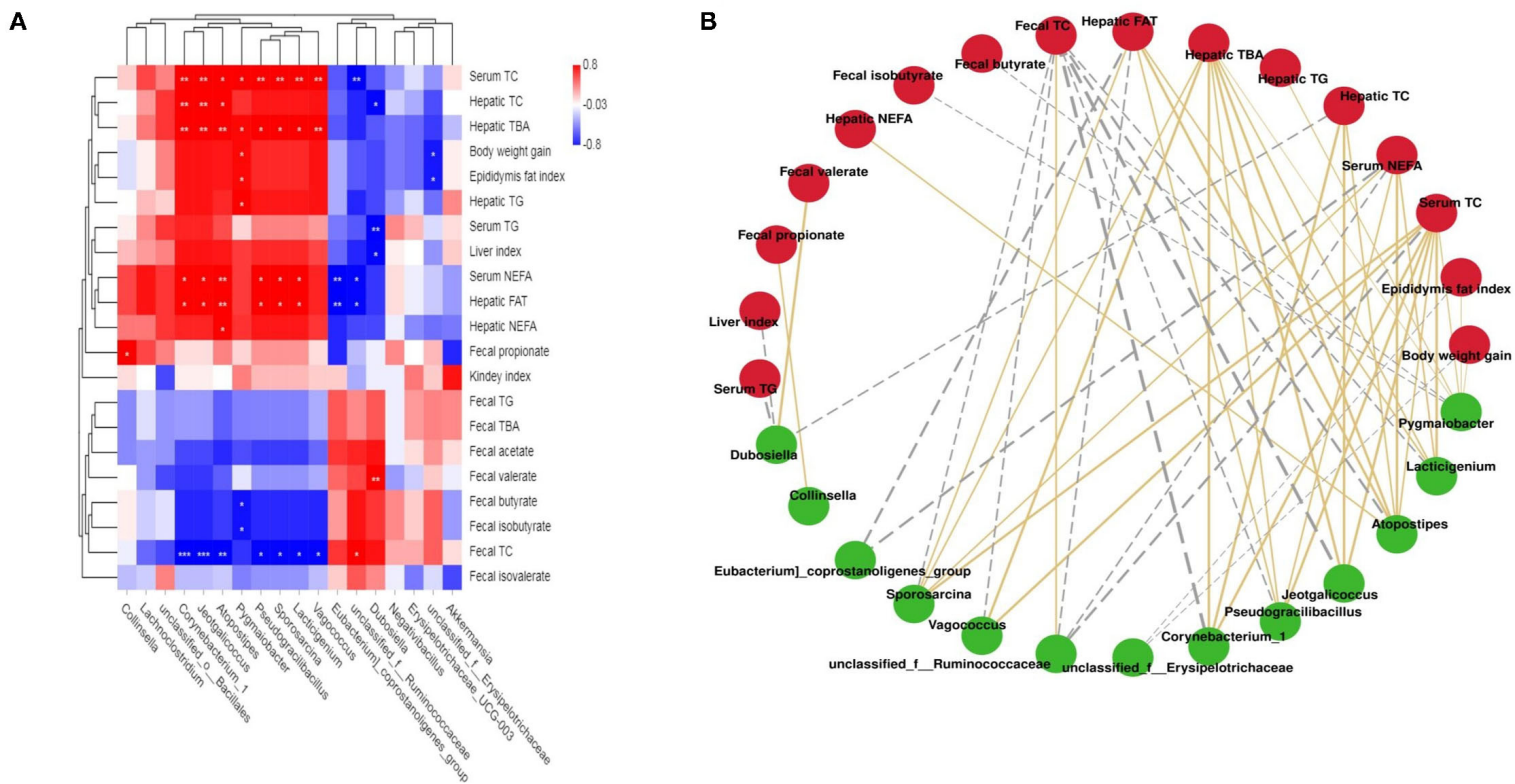
Statistical Spearman's correlations between the fecal microbiota of significant differences and lipid metabolic parameters. **(A)** Heatmap of Spearman's correlation. The intensity of the color represents the degree of association; **(B)** Correlation network constructed between bacterial species and microbial metabolic pathways. The edge width and color (gold: positive and gray: negative) are proportional to the correlation strength.

Correlations between lipid metabolic parameters and key intestinal microflora phylotypes were investigated using a network and heat map (Figure 6). Specifically, *Corynebacterum_1, Jeotgalicoccus, Atopostipes, Pygmaiobacter, Pscudgraililuls, Sporosarcina, Lacticigenium*, and *Vagococcus* were directly proportional to body weight gain, the liver index, epididymal fat, serum TC, TG, and NEFA levels, and hepatic TC, FAT, TG, TBA, and NEFA levels, but inversely related to fecal TC, TG, TBA, acetate, and butyrate levels. In addition, *unclassified_f_Ruminococcaceae, Eubacterium_coprostanoligenes_group*, and *Dubosiella* had a negative correlation with the liver index, serum NEFA, TG, and TC levels, and hepatic TBA, TC, TG, NEFA, and FAT levels, but a positive relationship with fecal TC, TG, TBA, acetate, and butyrate levels.

### Effects of LPLJ Supplementation on the Liver Metabolites in HFD-Fed Rats

**Figure 7:**
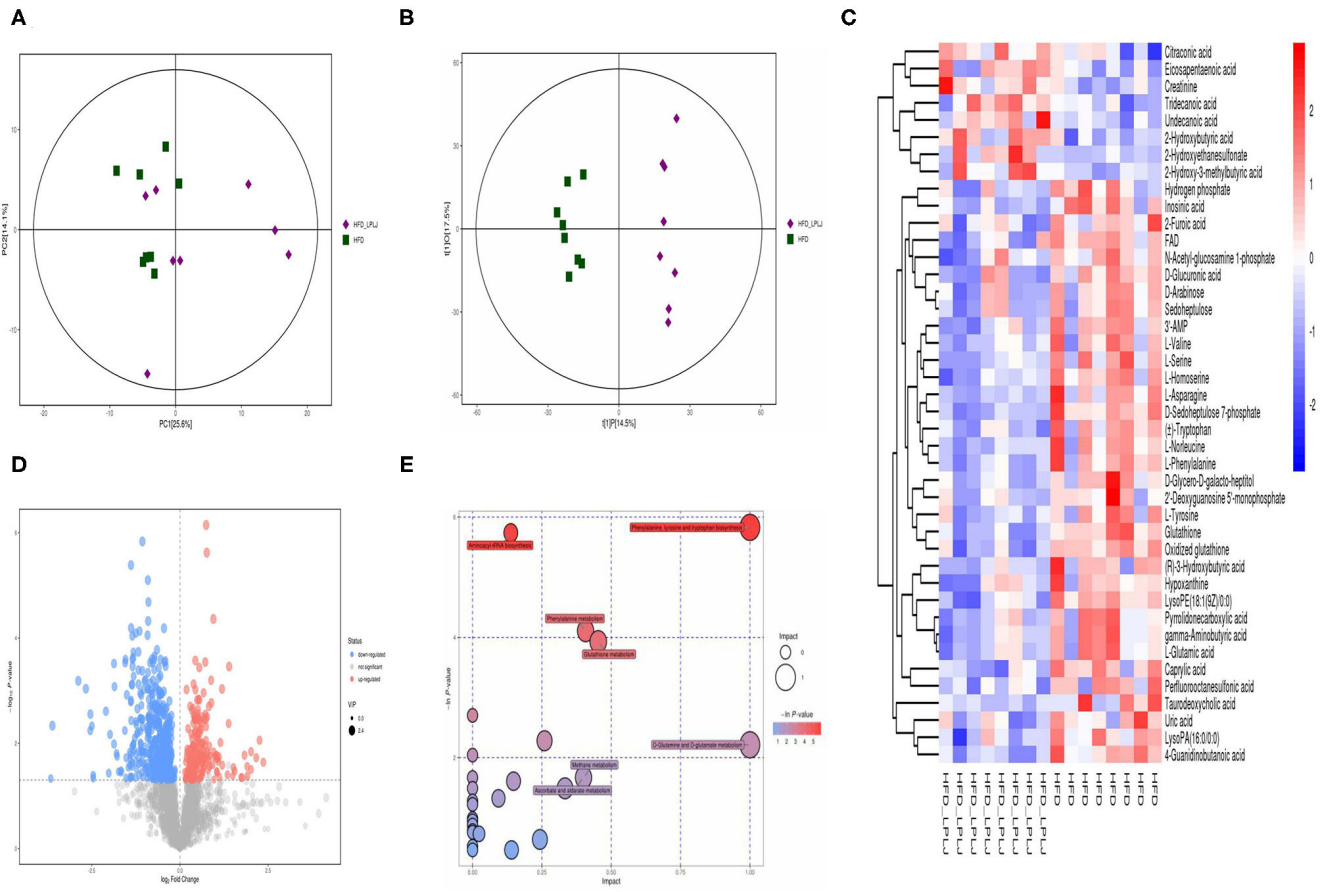
Liver metabolomic profiling by UPLC-QTOF/MS in negative model (ESI-) (*n* = 8 for each group). **(A)** PCA score plot for the HFD and LPLJ groups; **(B)** OPLS-DA score plot for the HFD and LPLJ groups; **(C)** Heatmap of relative abundance of significantly different metabolites (VIP > 1.0, and *p* < 0.05) between the HFD and LPLJ groups; **(D)** Permutation test from PLS-DA models, **(E)** The metabolic pathway impact prediction between the HFD and LPLJ groups in liver based on KEGG online database. The -ln(p) values from the pathway enrichment analysis are indicated on the horizontal axis, and the impact values are indicated on the vertical axis.

**Figure 8:**
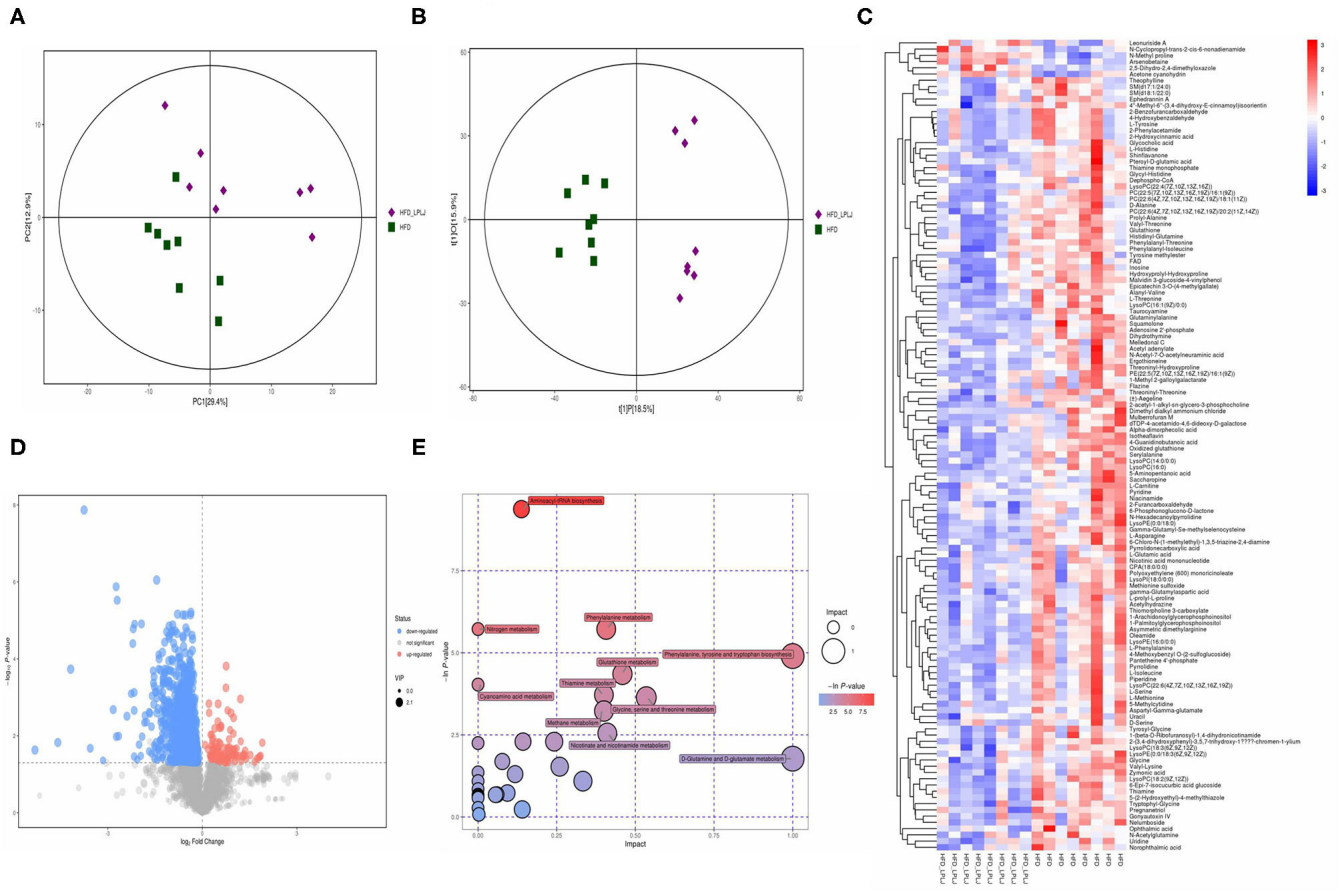
Liver metabolomic profiling by UPLC-QTOF/MS in positive model (ESI+) (*n* = 8 for each group). **(A)** PCA score plot for the HFD and LPLJ groups; **(B)** OPLS-DA score plot for the HFD and LPLJ groups; **(C)** Heatmap of relative abundance of significantly different metabolites (VIP > 1.0, and *p* < 0.05) between the HFD and LPLJ groups; **(D)** Permutation test from PLS-DA models, **(E)** The metabolic pathway impact prediction between the HFD and LPLJ groups in liver based on KEGG online database. The -ln(*p*) values from the pathway enrichment analysis are indicated on the horizontal axis, and the impact values are indicated on the vertical axis.

Principal component analysis (PCA) and orthogonal partial least-squares discrimination analysis (OPLS-DA) were used to observe the distinct changes in metabolite patterns in the liver. The results indicated that supplementation with LPLJ caused remarkable hepatic biochemical changes (Figures 7A,B, 8A,B). In the negative-ion mode, 42 potential biomarkers were identified. In the LPLJ group, 8 metabolites were upregulated, and 34 metabolites were downregulated compared with those in the HFD group. One hundred and thirty potential biomarkers were successfully identified between HFD and LPLJ groups in the positive ion mode (Figures 7C,D). The levels of six metabolites were increased and those of 124 metabolites were decreased in the LPLJ group compared with those in the HFD group (Figures 8C,D). To increase our understanding of metabolic changes after LPLJ supplementation in hyperlipidaemic rats, MetaboAnalyst 4.0 was used for the metabolic pathway enrichment analysis of differential liver metabolites (Figures 7E, 8E). The metabolic pathways in the negative-ion mode that were changed by LPLJ supplementation mainly included aminoacyl-tRNA biosynthesis, phenylalanine, tyrosine, and tryptophan biosynthesis, phenylalanine metabolism, glutathione metabolism, methane metabolism, D-glutamine and D-glutamate metabolism, and ascorbate and aldarate metabolism. In the positive ion mode, aminoacyl-tRNA biosynthesis, nitrogen metabolism, phenylalanine metabolism, glutathione metabolism, thiamine metabolism, cyanoamino acid metabolism, glycine, serine, and threonine metabolism, and methane, nicotinate, and nicotinamide metabolism were the primary metabolic pathways altered by LPLJ supplementation.

### Effects of LPLJ Supplementation on Hepatic MRNA Expression

**Figure 9:**
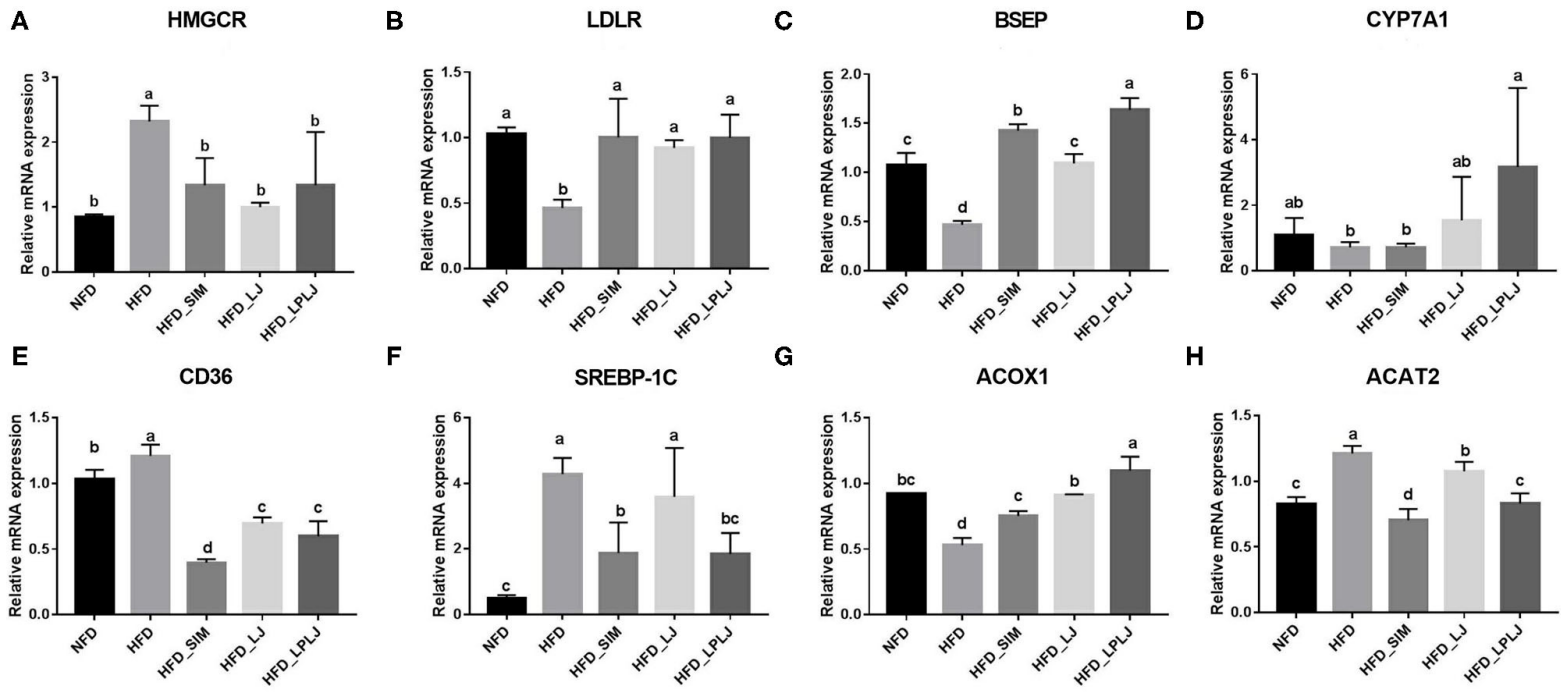
Effects of LPLJ consumption on the expression of hepatic related genes in HFD-fed rats. The bar graphs showed mRNA levels of **(A)** HMGCR, **(B)** LDLR, **(C)** BSEP, **(D)** CYP7A1, **(E)** CD36, **(F)** SREBP-1C, **(G)** ACOX1 and **(H)** ACAT2, which were determined by RT-qPCR. a-d, Mean values with different letters in the same column differ significantly (*p* < 0.05).

To identify the mechanisms underlying the moderating effect of LPLJ supplementation in rats fed an HFD, the liver mRNA expression levels of genes related to lipid metabolism were measured using qRT-PCR (Figure 9). HFD feeding increased the expression levels of cluster of differentiation 36 (CD36), recombinant acetyl coenzyme A acetyltransferase 2 (*ACAT2*), sterol regulatory element-binding protein-1c (SREBP-1c), and hydroxymethylglutaryl-CoA reductase (*HMGCR*) compared with those in the NFD group, and supplementation with LPLJ markedly reduced these abnormal expression levels (*p* < 0.05). On the contrary, the levels of bile salt export pump (*BSEP*), low-density lipoprotein receptor (*LDLR*), cholesterol 7α-hydroxylase (*CYP7A1*), and acyl-Coenzyme A oxidase 1 (*ACOX1*) in rats of the HFD group were reduced. Supplementation with LPLJ remarkably increased the mRNA levels of *LDLR, BESP, CYP7A1*, and *ACOX1* (*p* < 0.05).

## Discussion

Hyperlipidaemia has become an important public health problem worldwide (30). There is increasing evidence of diet interventions that have considered nutritional strategies for preventing or treating hyperlipidaemia (31–34). LJ is rich in nutrients and other diverse bioactive compounds, which have a variety of physiological functions, including reducing hyperlipidaemia (31, 32). Previous studies have shown that *Lactobacillus* can also regulate dyslipidaemia, the intestinal microbiome, and liver metabolism (33, 34). The results showed that dietary supplementation with LPLJ significantly prevent obesity, hyperlipidaemia, and NAFL disease (NAFLD) in HFD-fed rats. However, the exact regulatory mechanism of LPLJ that may reduce hyperlipidaemia and NAFLD requires further exploration.

In this study, HFD-fed rats showed increased weight gain, increased blood lipid levels, and liver lipid accumulation compared with those in the NFD group. Changes in serum biochemical indices can directly explain the metabolic status of the organism. Supplementation with LPLJ effectively reverses the increase in serum TC, TG, and NEFA levels in HFD-induced hyperlipidaemic rats, indicating that LPLJ may lower the risk of cardiovascular and atherosclerosis diseases (35). Moreover, oral supplementation with LPLJ reduces the fat indices of the liver, kidney, and epididymus, as well as reduces the adipocyte size in HFD-induced hyperlipidaemia. HFD promotes the abnormal accumulation of intrahepatic fat droplets, thereby leading to fatty liver. Using histopathological analysis, we also found a difference in liver lipid accumulation between the NFD and HFD group. The levels of lipid droplets in liver tissue were markedly decreased by dietary supplementation with LPLJ, indicating that it significantly improves liver lipid metabolism.

Hepatic lipid metabolism is one of the most critical factors in maintaining normal lipid metabolism *in vivo*. Cholesterol is usually absorbed from the small intestine after food intake, and then enters the enterohepatic circulation. It is transferred to the liver with BAs, and a small amount of BAs is excreted through the stool (36). BAs are commonly considered significant factors in the regulation of lipid and cholesterol metabolism. We found that dietary supplementation with LPLJ markedly increased the levels of hepatic BAs and excretion of fecal BAs. Moreover, the levels of TC and TG in the liver were similar to the serum biochemical parameters, which are consistent with the current study (37). We speculated that LPLJ supplementation may promote the excretion of BAs and lower the lipid level in serum and accumulation in the liver.

To further investigate the regulatory mechanism of LPLJ dietary supplementation for hyperlipidaemia related to NAFLD, liver metabolomics was used in this study. The results showed that supplementation with LPLJ mainly relieved hyperlipidaemia by regulating the pathways involved in aminoacyl-tRNA biosynthesis, phenylalanine, tyrosine, and tryptophan biosynthesis, as well as those involved in phenylalanine, glutathione, and D-glutamine, and D-glutamate metabolism. It is worth noting that phenylalanine, tyrosine, and tryptophan are essential amino acids in the human body. Tyrosine, which is inversely associated with the risk of obesity, is synthesized from phenylalanine in a reaction catalyzed by phenylalanine hydroxylase, and affects the metabolism of glucose and lipids in the body (38). Glutamate metabolism is important in the regulation of protein and nucleic acid biosynthesis. Glutamine is a non-essential amino acid that participates in central metabolic processes as a substrate for the tricarboxylic acid cycle (39). Petrus et al. found that glutamine reduces inflammation in adipose tissue in rats and is inversely associated with fat mass (40). Our results show that dietary supplementation with LPLJ ameliorates dyslipidaemia in hyperlipidaemic rats by affecting these metabolic pathways.

The intestinal flora has a significant influence on the treatment of hyperlipidaemia (41, 42). Previous research has shown that a variety of bioactive components in seaweeds can regulate the microecology of gut microbiota (43). High-throughput sequencing showed that compared with that in the NFD group, the relative abundance of 15 key microbial phylotypes were altered in HFD-fed rats, indicating that a gut microbial disorder occurred in HFD-induced hyperlipidaemic rats. However, supplementation with LPLJ distinctly regulated the gut microbiota in HFD-fed rats, including modulating the relative abundance of functionally related microbial system types. Compared with that in the NFD group, the relative abundances of *Collinsella* and *Lachnoclostridium* in the HFD group significantly increased. *Collinsella* is associated with obesity and atherosclerosis in human studies (44–46). Another study has shown that the relative abundance of *Collinsella* is significantly elevated in patients with non-alcoholic steatohepatitis (47). In addition, it has been reported that *Lachnoclostridium* is significantly positively associated with TGs and TC (48). However, dietary supplementation with LPLJ reversed the abnormal elevation of the relative abundance of *Collinsella* and *Lachnoclostridium*. In the current study, we speculated that compared with that in the HFD group, the abundance of *Collinsella* and *Lachnoclostridium* increased in the LPLJ group to exert lipid-lowering effects. Notably, the relative abundance of *Akkermansia* and *unclassified _f_Erysipelotrichaceae* was dramatically increased by dietary supplementation with LPLJ. As the most abundant probiotic member of the human gut, the abundance of *Akkermansia* is negatively correlated with obesity (49). Growing evidence shows that *Akkermansia* can ameliorate intestinal dysbiosis caused by HFD (50). A previous study has shown that the relative abundance of *unclassified _f_Erysipelotrichaceae* could also be increased to reduce abnormal lipid metabolism (51). These results suggest that this is one of the mechanisms by which LPLJ regulates hyperlipidaemia. Correlation analysis results using heat map and network showed that the types of intestinal microbial systems are closely correlated with lipid metabolism parameters. Supplementation with LPLJ decreased the relative abundance of *Corynebacterium_1, Jeotgalcoccus*, and *Atopostipes*, and exhibited negative relationships with fecal TG, TC, and TBA levels and the metabolism of SCFAs, but was positively associated with serum TC, TG, TBA, and NEFA levels, hepatic TG, TC, TBA, and FAT levels, body weight gain, and epididymal fat index. This association is thought to be related to effects on the lipid metabolism and the diet of the host. These results showed that dietary supplementation with LPLJ can improve the intestinal microbiota disturbance caused by the HFD, thereby alleviating obesity and lipid abnormalities in the liver.

Moreover, the expression of mRNA genes related to adipogenesis, fatty acid oxidation, and BA production was analyzed to evaluate the effect of LPLJ intervention on cholesterol and lipid metabolism. Compared with the NFD group, HFD feeding markedly increased mRNA levels of liver genes encoding HMGCR, CD36, SREBP-1c, and ACAT2, whereas LPLJ intervention significantly lowered these abnormal transcription levels. Among them, SREBP-1c plays a significant role in adjusting the expression of adipogenic genes involved in liver fatty acid synthesis (52). CD36 plays an important role in transmembrane transport and energy metabolism of long-chain fatty acids (53). Previous experiments have indicated that ACAT2 inhibits TG synthesis by decreasing the transition of esterified cholesterol to cholesterol ester, which is consistent with the results of the current study that dietary supplementation with LPLJ notably reduced the level of ACAT2 in the liver (54). The present study also found that HFD increased the level of TC in the liver by increasing HMGCR expression, and these findings were consistent with those of a previous study (55). However, HMGCR expression was significantly reduced after dietary supplementation with LPLJ. Conversely, the mRNA levels of LDLR, BSEP, CYP7A1, and ACOX1 in rats of the HFD group were lower than those in rats in the NFD group. Supplementation with LPLJ remarkably increased the expression of LDLR, BSEP, CYP7A1, and ACOX1. Notably, the expression of BA synthetic genes, such as CYP7A1, was significantly increased, and that of BSEP, a transporter of BAs, was also increased, which was consistent with Kwon's findings (56). In addition, ACOX1 is involved in the β-oxidative activation of fatty acids (57). BA, an endogenous signaling molecule synthesized by liver cholesterol, affects insulin secretion and the regulation of glucose and lipid metabolism. The results of this study show that dietary supplementation with LPLJ significantly upregulated the mRNA levels of genes related to BA homeostasis and decreased hepatic BA levels that were increased by HFD.

In conclusion, we found that dietary supplementation with LPLJ has the potential to modulate hyperlipidaemia in HFD-fed rats, by reducing serum and liver lipid levels, as well as higher fecal lipid and SCFA levels. In addition, the regulatory effect of LPLJ intervention on hyperlipidaemia may be related to the significant changes in liver metabolites, genes related to liver lipid metabolism, and intestinal microbiota. However, this study was limited by the lack of a group with HFD and *L. plantarum FZU3013* supplementation. Thus, further research is required to identify the functional components of LPLJ that are essential for regulating lipid metabolism.

## Data Availability Statement

The original contributions presented in the study are included in the article/supplementary material, further inquiries can be directed to the corresponding authors.

## Ethics Statement

The animal study was reviewed and approved by Institutional Animal Care and Use Committee of College of Food Science, Fujian Agriculture and Forestry University.

## Author Contributions

J-PH, W-JC, and PL contributed to conception and design of the study. T-TZ, B-FZ, and M-LW performed the statistical analysis. J-PH wrote the first draft of the manuscript. T-TZ, B-FZ, RS, YZ, and L-JC wrote sections of the manuscript. All authors contributed to manuscript revision, read, and approved the submitted version.

## Funding

This study was supported by the Innovative Scientific Research Program of Fujian Agriculture and Forestry University (Grant nos. CXZX2018066 and CXZX2017017).

## Conflict of Interest

The authors declare that the research was conducted in the absence of any commercial or financial relationships that could be construed as a potential conflict of interest.

## Publisher's Note

All claims expressed in this article are solely those of the authors and do not necessarily represent those of their affiliated organizations, or those of the publisher, the editors and the reviewers. Any product that may be evaluated in this article, or claim that may be made by its manufacturer, is not guaranteed or endorsed by the publisher.
